# CAREGIVER USABILITY TESTING OF WEB-BASED AND VIRTUAL REALITY REMINISCENCE THERAPY FOR PERSONS WITH DEMENTIA

**DOI:** 10.1093/geroni/igac059.2821

**Published:** 2022-12-20

**Authors:** Winnie Sun, Alvaro Quevedo

**Affiliations:** Ontario Tech University, Oshawa, Ontario, Canada; Ontario Tech University, Oshawa, Ontario, Canada

## Abstract

Reminiscence therapy (RT) is a multi-sensory treatment that uses a combination of sight, touch, taste, smell and sound to help persons with dementia (PWD) remember events, people and places from their past lives. Currently, digital technologies such as mobile applications and immersive solutions including virtual and augmented reality, are gaining momentum as supplementary tools for RT. This paper presents a usability study of a web-based and virtual reality application to understand the limitations and opportunities of digital platforms for facilitating engaging experiences for PWD towards recalling memories, while easing the therapy process for the caregivers. A total of ten healthcare caregivers were recruited from the Geriatric Dementia Unit and Geriatric Transitional Unit in Ontario Shores Center for Mental Health Sciences, Ontario Canada. Usability feedback from the caregivers were collected from the interviews after the completion of the System Usability Scale (SUS) questionnaire. Institutional caregivers found both web-based and virtual reality (VRRT) usable with SUS score above average (68/100), but required improvements related to the onboarding training of caregivers. The interview revealed four overarching themes related to the VRRT: (1) Ease of use; (2) Positive impact on caregiving; (3) Potential of reduction in responsive behaviors; (4) Feasibility for promoting social connection during COVID-19 pandemic. Next steps will focus on improving the user experience and expanding the application for immersive VR supporting head-mounted displays, hand tracking, and physiological measures, as well as conducting an usability study with PWD to expand our understanding of using RT digital tools with various levels of immersion.

